# Supplementary material for: Probiotic-conditioned microbiota from preterm infants modulate immune response to pathogen challenge in a microbiota-humanized mouse model
Source: Front Immunol. 2026 Apr 20;17:1761680. doi: 10.3389/fimmu.2026.1761680 (PMC13135994; doi:10.3389/fimmu.2026.1761680)
Supplement: Supplementary file 1 [file DataSheet1.pdf]

Supplementary Material

Probiotic-conditioned microbiota from preterm infants modulate immune response to pathogen challenge in a microbiota-humanized mouse model

Justine Smout, Till-Robin Lesker, Lisa Hoenicke, Diego Ortiz, Mangge Zou, Friederike Kruse, Sabine Pirr, Maike Willers, Christoph Härtel, Christine Falk, Natalia Torow, Dorothee Viemann, Till Strowig, Jochen Huehn

Supplementary Figures

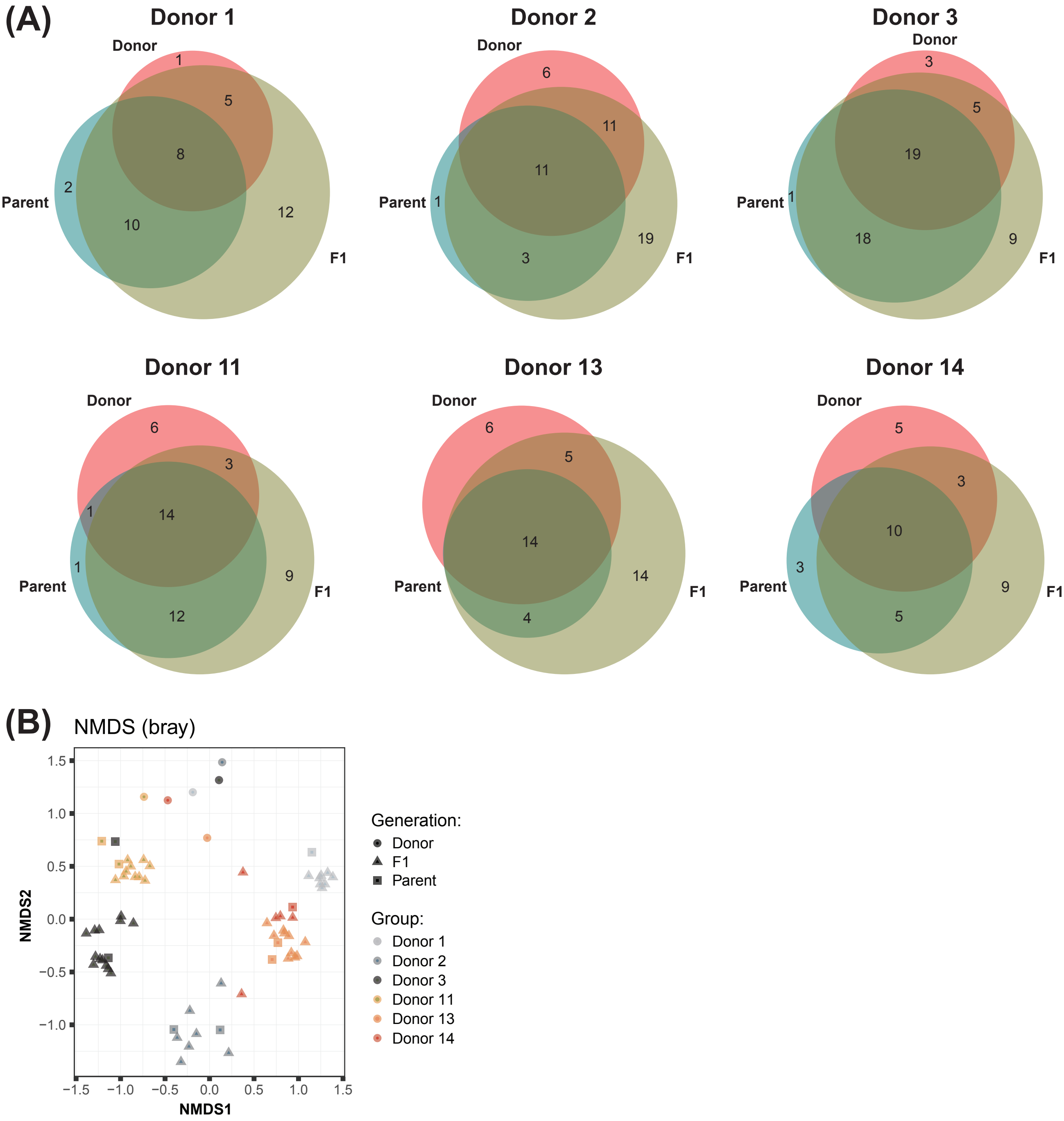

Figure S1. Colonization of mice with different preterm microbiota. (A) Venn diagrams displaying shared species between the donor (red), the parent population (teal) and the F1 generation (olive). **(B)** β-diversity of fecal samples from donor, parent and F1 generation was analyzed using the Bray-Curtis dissimilarity matrix and NMDS. Results are pooled from 1 (Donors 1, 11, 13, 14) or 2 (Donors 2, 3) independent experiments.

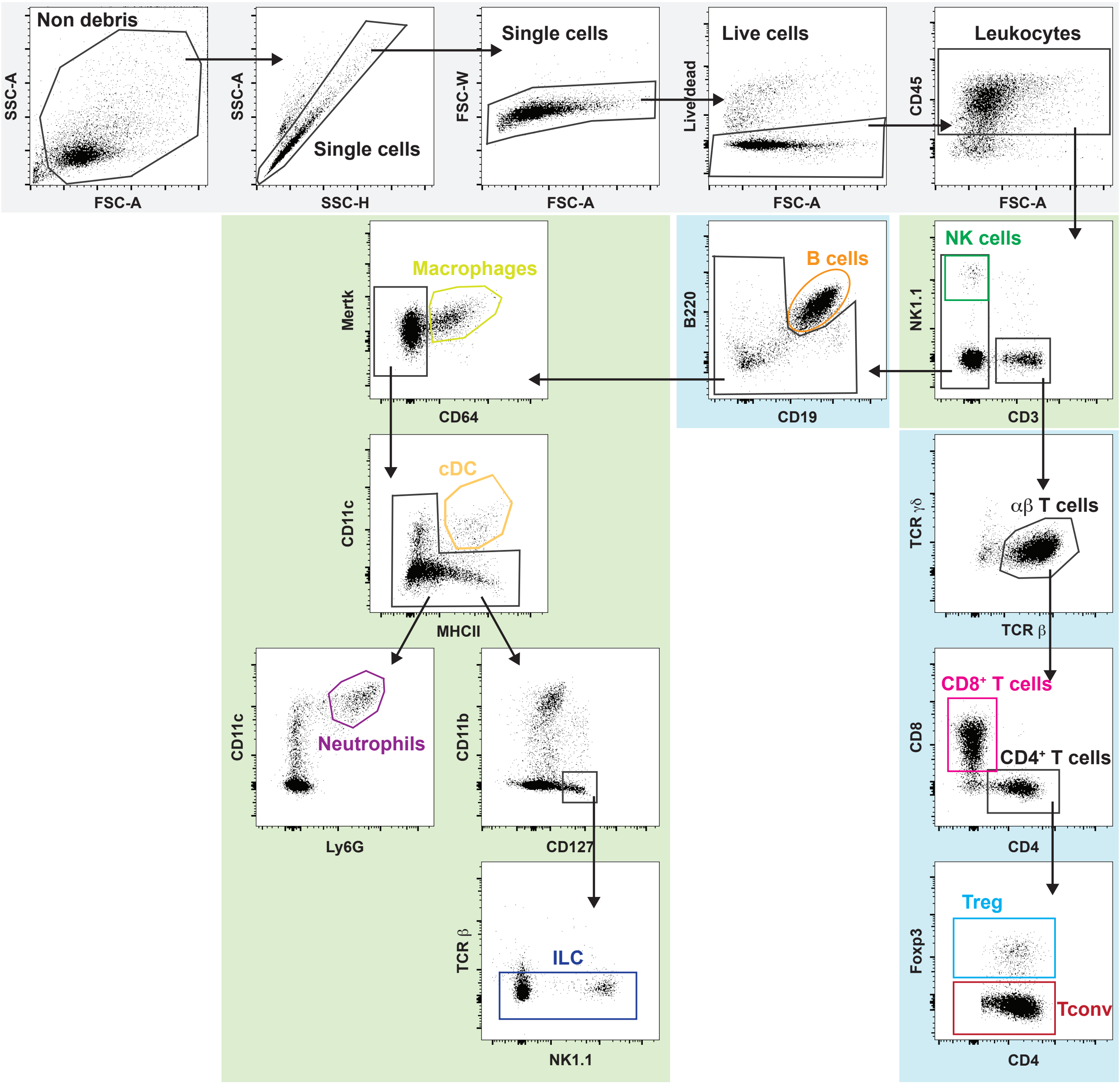

Figure S2. Gating strategy. Representative dot plots to identify the major innate (green box; natural killer (NK) cells, macrophages, conventional dendritic cells (cDC), neutrophils, and innate lymphoid cells (ILC)) and adaptive (blue box, B cells, CD8^{+} T cells, regulatory T cells (Treg), and conventional T cells (Tconv)) immune cells, shown here for 3-week-old spleen.

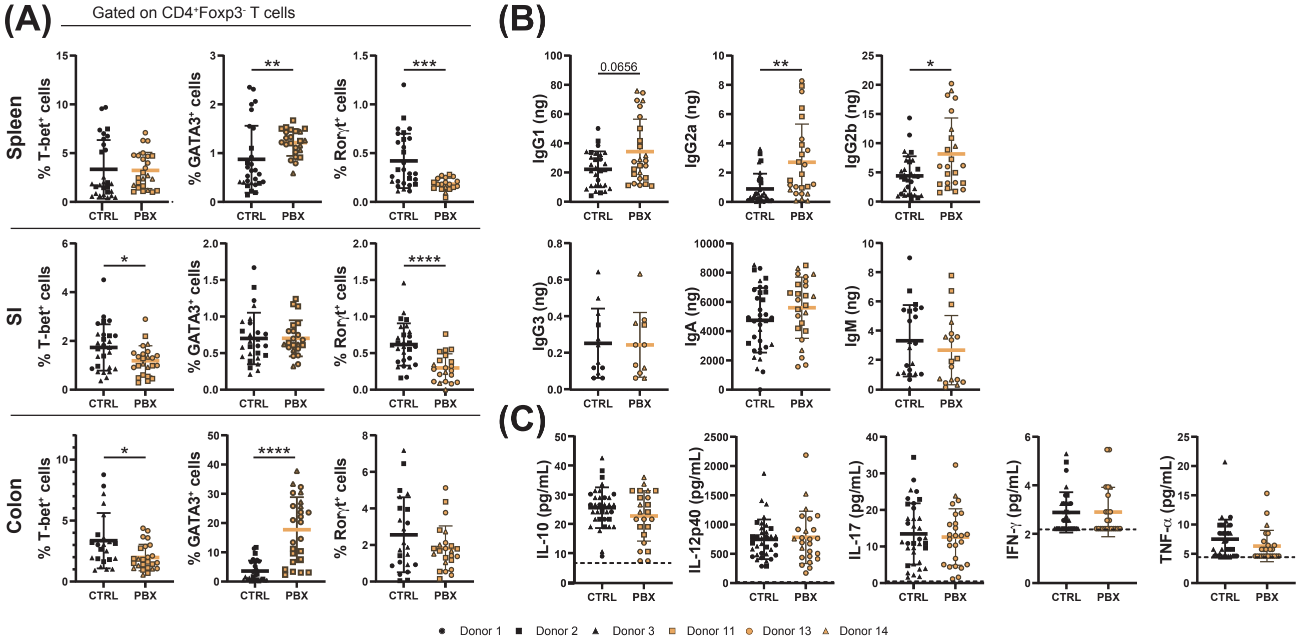

Figure S3. Probiotic-induced microbiota skews T helper differentiation and triggers a selective mucosal IgG subclasses elevation in the absence of systemic inflammation. Spleen, small intestine (SI), colon, cecal content and serum were collected from 3-week-old neonates born to mothers microbiota-humanized with either placebo (CTRL) or probiotic (PBX)-treated preterm fecal microbiota. The organs samples were analyzed by flow cytometry while the cecal contents and serum were analyzed by multiplex assay. **(A)** Percentages of CD4$^+$ conventional T cells (Tconv) expressing the transcription factors associated with Th1 (T-bet) or Th17 (Rorγt). **(B)** Amount of IgG1, IgG2a, IgG2b, IgG3, IgA, and IgM in the total cecal content. **(C)** Concentration of serum cytokines IL-10, IL-12p40, IL-17A, IFN-γ, and TNF-α. The dashed line represents the limit of detection (LOD). Results are pooled from 1 (Donors 1, 11, 13, 14) or 2 (Donors 2, 3) independent experiments and are presented as mean ± SD. Each symbol represents one mouse: CTRL donors - Donor 1 (●, n=8), Donor 2 (■, n=7), Donor 3 (▲, n=13); PBX donors - Donor 11 (□, n=10), Donor 13 (○, n=10), Donor 14 (△, n=5) (* = p<0.05; ** = p<0.005; *** = p<0.001; **** = p <0.0001).

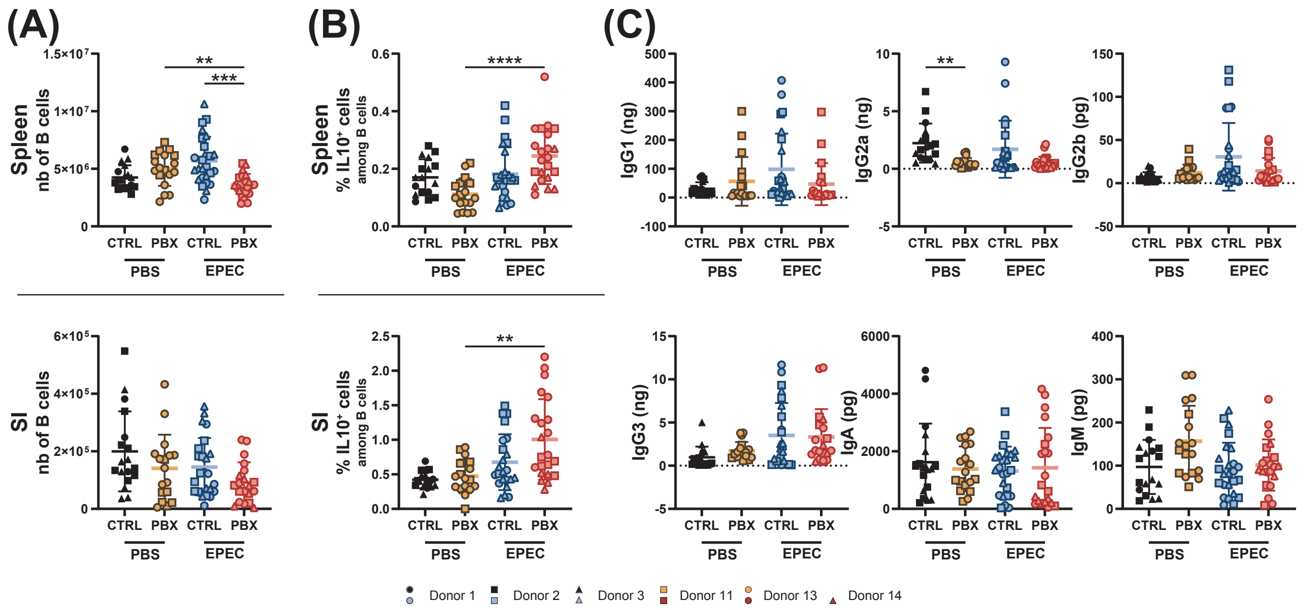

Figure S4. Effect of probiotic-conditioned microbiota on B cells during early life gastrointestinal infection. Spleen and small intestine (SI) of sham (PBS) or infected (EPEC) neonates at postnatal day 12 born to mothers microbiota-humanized with placebo (CTRL) or probiotic (PBX) treated preterm feces were collected 7 days post-infection and analyzed by flow cytometry. **(A)** Cell counts of B cells. **(B)** Parental percentage of regulatory (IL-10^{+}) B cells. **(C)** Amount of IgG1, IgG2a, IgG2b, IgG3, IgA, and IgM in the total cecal content. Results are pooled from 1 (Donors 1, 11, 14), 2 (Donors 2, 13) or 3 (Donor 3) independent experiments and are presented as mean ± SD. Each symbol represents one mouse. Sample sizes per donor are given as *n*PBS/*n*EPEC: CTRL donors - Donor 1 (●, 3/7), Donor 2 (■, 8/9), Donor 3 (▲, 7/10); PBX donors - Donor 11 (□, 7/5), Donor 13 (○, 11/11), Donor 14 (△, 0/6) (** = $p<0.005$; *** = $p<0.001$; **** = $p<0.0001$).
